# Robust Estimation for the Single Index Model Using Pseudodistances

**DOI:** 10.3390/e20050374

**Published:** 2018-05-17

**Authors:** Aida Toma, Cristinca Fulga

**Affiliations:** 1 Department of Applied Mathematics, Bucharest Academy of Economic Studies, 010374 Bucharest, Romania; 2 “Gh. Mihoc - C. Iacob” Institute of Mathematical Satistics and Applied Mathematics, Romanian Academy, 010071 Bucharest, Romania

**Keywords:** minimum divergence methods, robustness, single index model

## Abstract

For portfolios with a large number of assets, the single index model allows for expressing the large number of covariances between individual asset returns through a significantly smaller number of parameters. This avoids the constraint of having very large samples to estimate the mean and the covariance matrix of the asset returns, which practically would be unrealistic given the dynamic of market conditions. The traditional way to estimate the regression parameters in the single index model is the maximum likelihood method. Although the maximum likelihood estimators have desirable theoretical properties when the model is exactly satisfied, they may give completely erroneous results when outliers are present in the data set. In this paper, we define minimum pseudodistance estimators for the parameters of the single index model and using them we construct new robust optimal portfolios. We prove theoretical properties of the estimators, such as consistency, asymptotic normality, equivariance, robustness, and illustrate the benefits of the new portfolio optimization method for real financial data.

## 1. Introduction

The problem of portfolio optimization in the mean-variance approach depends on a large number of parameters that need to be estimated on the basis of relatively small samples. Due to the dynamics of market conditions, only a short period of market history can be used for estimation of the model’s parameters. In order to reduce the number of parameters that need to be estimated, the single index model proposed by Sharpe (see [1,2]) can be used. The traditional estimators for parameters of the single index model are based on the maximum likelihood method. These estimators have optimal properties for normally distributed variables, but they may give completely erroneous results in the presence of outlying observations. Since the presence of outliers in financial asset returns is a frequently occurring phenomenon, robust estimates for the parameters of the single index model are necessary in order to provide robust and optimal portfolios.

Our contribution to robust portfolio optimization through the single index model is based on using minimum pseudodistance estimators.

The interest on statistical methods based on information measures and particularly on divergences has grown substantially in recent years. It is a known fact that, for a wide variety of models, statistical methods based on divergence measures have some optimal properties in relation to efficiency, but especially in relation to robustness, representing viable alternatives to the classical methods. We refer to the monographs of Pardo [3] and Basu et al. [4] for an excellent presentation of such methods, for their importance and applications.

We can say that the minimum pseudodistance methods for estimation go to the same category as the minimum divergence methods. The minimum divergence estimators are defined by minimizing some appropriate divergence between the assumed theoretical model and the true model corresponding to the data. Depending on the choice of the divergence, minimum divergence estimators can afford considerable robustness with a minimal loss of efficiency. The classical minimum divergence methods require nonparametric density estimation, which imply some difficulties such as the bandwidth selection. In order to avoid the nonparametric density estimation in minimum divergence estimation methods, some proposals have been made in [5,6,7] and robustness properties of such estimators have been studied in [8,9].

The pseudodistances that we use in the present paper were originally introduced in [6], where they are called "type-0" divergences, and corresponding minimum divergence estimators have been studied. They are also obtained (using a cross entropy argument) and extensively studied in [10] where they are called γ-divergences. They are also introduced in [11] in the context of decomposable pseudodistances. By its very definition, a pseudodistance satisfies two properties, namely the nonnegativity and the fact that the pseudodistance between two probability measures equals to zero if and only if the two measures are equal. The divergences are moreover characterized by the information processing property, i.e., by the complete invariance with respect to statistically sufficient transformations of the observation space (see [11], p. 617). In general, a pseudodistance may not satisfy this property. We adopted the term pseudodistance for this reason, but in the literature we can also meet the other terms above. The minimum pseudodistance estimators for general parametric models have been presented in [12] and consist of minimization of an empirical version of a pseudodistance between the assumed theoretical model and the true model underlying the data. These estimators have the advantages of not requiring any prior smoothing and conciliate robustness with high efficiency, usually requiring distinct techniques.

In this paper, we define minimum pseudodistance estimators for the parameters of the single index model and using them we construct new robust optimal portfolios. We study properties of the estimators, such as, consistency, asymptotic normality, robustness and equivariance and illustrate the benefits of the proposed portfolio optimization method through examples for real financial data.

We mention that we define minimum pseudodistance estimators, and prove corresponding theoretical properties, for the parameters of the simple linear regression model (35), associated with the single index model. However, in a very similar way, we can define minimum pseudodistance estimators and obtain the same theoretical results for the more general linear regression model $Y_{j}=X_{j}^{T}\beta +e_{j}$, j=1,…,n, where the errors $e_{j}$ are i.i.d. normal variables with mean zero and variance $\sigma^{2}$, $X_{j}={\left(X_{j1},\ldots ,X_{jp}\right)}^{T}$ is the vector of independent variables corresponding to the *j*-th observation and $\beta ={\left(\beta_{1},\ldots ,\beta_{p}\right)}^{T}$ represents the regression coefficients.

The rest of the paper is organized as follows. In Section 2, we present the problem of robust estimation for some portfolio optimization models. In Section 3, we present the proposed approach. We define minimum pseudodistance estimators for regression parameters corresponding to the single index model and obtain corresponding estimating equations. Some asymptotic properties and equivariance properties of these estimators are studied. The robustness issue for estimators is considered through the influence function analysis. Using minimum pseudodistance estimators, new optimal portfolios are defined. Section 4 presents numerical results illustrating the performance of the proposed methodology. Finally, the proofs of the theorems are provided in the Appendix A.

## 2. The Single Index Model

Portfolio selection represents the problem of allocating a given capital over a number of available assets in order to maximize the return of the investment while minimizing the risk. We consider a portfolio formed by a collection of *N* assets. The returns of the assets are given by the random vector $X:={\left(X_{1},\ldots ,X_{N}\right)}^{T}$. Usually, it is supposed that *X* follows a multivariate normal distribution $N_{N}\left(\mu ,\Sigma \right)$, with μ being the vector containing the mean returns of the assets and $\Sigma =(\sigma_{ij})$ the covariance matrix of the assets returns. Let $w:={\left(w_{1},\ldots ,w_{N}\right)}^{T}$ be the vector of weights associated with the portfolio, where $w_{i}$ is the proportion of capital invested in the asset *i*. Then, the total return of the portfolio is defined by the random variable

(1) $$w^{T}X=w_{1}X_{1}+\cdots +w_{N}X_{N}.$$

The mean and the variance of the portfolio return are given by

(2) $$\begin{array}{ccc} R(w) & := & w^{T}\mu , \end{array}$$

(3) $$\begin{array}{ccc} S(w) & := & w^{T}\Sigma w. \end{array}$$

A classical approach for portfolio selection is the mean-variance optimization introduced by Markowitz [13]. For a given investor’s risk aversion λ>0, the mean-variance optimization gives the optimal portfolio $w^{*}$, solution of the problem

(4) $$\arg\max_{w}\left\{R\left(w\right)-\frac{\lambda }{2}S\left(w\right)\right\},$$

with the constraint $w^{T}e_{N}=1$, $e_{N}$ being the *N*-dimensional vector of ones. The solution of the optimization problem (4) is explicit, the optimal portfolio weights for a given value of λ being

(5) $$w^{*}=\frac{1}{\lambda }\Sigma^{-1}\left(\mu -\eta e_{N}\right),$$

where

(6) $$\eta =\frac{e_{N}^{T}\Sigma^{-1}\mu -\lambda }{e_{N}^{T}\Sigma^{-1}e_{N}}.$$

This is the case when short selling is allowed. When short selling is not allowed, we have a supplementary constraint in the optimization problem, namely all the weights $w_{i}$ are positive.

Another classical approach for portfolio selection is to minimize the portfolio risk defined by the portfolio variance, under given constraints. This means determining the optimal portfolio $w^{*}$ as a solution of the optimization problem

(7) $$\arg\min_{w}S\left(w\right),$$

subject to $R\left(w\right)=w^{T}\mu \geq \mu_{0}$, for a given value $\mu_{0}$ of the portfolio return.

However, the mean-variance analysis has been criticized for being sensitive to estimation errors of the mean and the covariance of the assets returns. For both optimization problems above, estimations of the input parameters μ and Σ are necessary. The quality and hence the usefulness of the results of the portfolio optimization problem critically depend on the quality of the statistical estimates for these input parameters. The mean vector and the covariance matrix of the returns are in practice estimated by the maximum likelihood estimators under the multivariate normal assumption. When the model is exactly satisfied, the maximum likelihood estimators have optimal properties, being the most efficient. On the other hand, in the presence of outlying observations, these estimators may give completely erroneous results and consequently the weights of the corresponding optimal portfolio may be completely misleading. It is a known fact that outliers frequently occur in asset returns, where an outlier is defined to be an unusually large value well separated from the bulk of the returns. Therefore, robust alternatives to the classical approaches need to be carefully analyzed.

For an overview on the robust methods for portfolio optimization, using robust estimators of the mean and covariance matrix in the Markowitz’s model, we refer to [14]. We also cite the methods proposed by Vaz-de Melo and Camara [15], Perret-Gentil and Victoria-Feser [16], Welsch and Zhou [17], DeMiguel and Nogales [18], and Toma and Leoni-Aubin [19].

On the other hand, in portfolio analysis, one is sometimes faced with two conflicting demands. Good quality statistical estimates require a large sample size. When estimating the covariance matrix, the sample size must be larger than the number of different elements of the matrix. For example, for a portfolio involving 100 securities, this would mean observations from 5050 trading days, which is about 20 years. From a practical point of view, considering such large samples is not adequate for the considered problem. Since the market conditions change rapidly, very old observations would lead to irrelevant estimates for the current or future market conditions. In addition, in some situations, the number of assets could even be much larger than the sample size of exploitable historical data. Therefore, estimating the covariance matrix of asset returns is challenging due to the high dimensionality and also to the heavy-tailedness of asset return data. It is a known fact that extreme events are typical in financial asset prices, leading to heavy-tailed asset returns. One way to treat these problems is to use the single index model.

The single index model (see [1]) allows us to express the large number of covariances between the returns of the individual assets through a significantly smaller number of parameters. This is possible under the hypothesis that the correlation between two assets is strictly given by their dependence on a common market index. The return of each asset *i* is expressed under the form

(8) $$X_{i}=\alpha_{i}+\beta_{i}X_{M}+e_{i},$$

where $X_{M}$ is the random variable representing the return of the market index, $e_{i}$ are zero mean random variables representing error terms and $\alpha_{i},\beta_{i}$ are new parameters to be estimated. It is supposed that the $e_{i}$’s are independent and also that the $e_{i}$s are independent of $x_{M}$. Thus, $E(e_{i})=0$, $E(e_{i}e_{j})=0$ and $E(e_{i}x_{M})=0$ for all *i* and all j≠i.

The intercept in Equation (35) represents the asset’s expected return when the market index return is zero. The slope coefficient $\beta_{i}$ represents the asset’s sensitivity to the index, namely the impact of a unit change in the return of the index. The error $e_{i}$ is the return variation that cannot be explained by the index.

The following notations are also used:

$$\begin{array}{ccc} \sigma_{i}^{2} & := & Var\left(e_{i}\right),\;\;\;\mu_{M}:=E\left(X_{M}\right),\;\;\;\sigma_{M}^{2}:=Var\left(X_{M}\right). \end{array}$$

Using Equation (35), the components of the parameters μ and Σ from the models (4) and (7) are given by

(9) $$\begin{array}{ccc} \mu_{i} & = & \alpha_{i}+\beta_{i}\mu_{M}, \end{array}$$

(10) $$\begin{array}{ccc} \sigma_{ii} & = & \beta_{i}^{2}\sigma_{M}^{2}+\sigma_{i}^{2}, \end{array}$$

(11) $$\begin{array}{ccc} \sigma_{ij} & = & \beta_{i}\beta_{j}\sigma_{M}^{2}. \end{array}$$

Both variances and covariances are determined by the assets’ betas and sigmas and by the standard deviation of the market index. Thus, the N(N+1)/2 different elements of the covariance matrix Σ can be expressed by 2N+1 parameters $\beta_{i},\sigma_{i}$, $\sigma_{M}$. This is a significant reduction of the number of parameters that need to be estimated.

The traditional estimators for parameters of the single index model are based on the maximum likelihood method. These estimators have optimal properties for normally distributed variables, but they may give completely erroneous results in the presence of outlying observations. Therefore, robust estimates for the parameters of the single index model are necessary in order to provide robust and optimal portfolios.

## 3. Robust Estimators for the Single Index Model and Robust Portfolios

### 3.1. Definitions of the Estimators

Consider the linear regression model

(12) $$X=\alpha +\beta X_{M}+e.$$

Suppose we have i.i.d. two-dimensional random vectors $Z_{j}=\left(X_{Mj},X_{j}\right)$, j=1,…,n, such that $X_{j}=\alpha +\beta X_{Mj}+e_{j}$. The random variables $e_{j}$, j=1,…,n, are i.i.d. with N(0,σ) and independent on the $X_{Mj}$, j=1,…,n.

The classical estimators for the unknown parameters α,β,σ of the linear regression model are the maximum likelihood estimators (MLE). The classical MLE estimators perform well if the model hypotheses are satisfied exactly and may otherwise perform poorly. It is well known that the MLE are not robust, since a small fraction of outliers, even one outlier may have an important effect inducing significant errors on the estimates. Therefore, robust alternatives of the MLE should be considered, in order to propose robust estimates for the single index model, leading then to robust portfolio weights.

In order to robustly estimate the unknown parameters α,β,σ, suppressing the outsized effects of outliers, we use the approach based on pseudodistance minimization.

For two probability measures P,Q admitting densities *p*, respectively, *q* with respect to the Lebesgue measure, we consider the following family of pseudodistances (also called γ-divergences in some articles) of orders γ>0

(13) $$R_{\gamma }\left(P,Q\right):=\frac{1}{\left(1+\gamma \right)}\ln\left(\int p^{\gamma }dP\right)+\frac{1}{\gamma (1+\gamma )}\ln\left(\int q^{\gamma }dQ\right)-\frac{1}{\gamma }\ln\left(\int p^{\gamma }dQ\right),$$

satisfying the limit relation

$$R_{\gamma }\left(P,Q\right)\to R_{0}\left(P,Q\right):=\int \ln\frac{q}{p}dQ\;\;\mathrm{for}\;\;\gamma ↓0.$$

Note that $R_{0}\left(P,Q\right)$ is the well-known modified Kullback–Leibler divergence. Minimum pseudodistance estimators for parametric models, using the family (13), have been studied by [6,10,11]. We also mention that pseudodistances (13) have also been used for defining optimal robust M-estimators with the Hampel’s infinitesimal approach in [20].

For the linear regression model, we consider the joint distribution of the entire data, the explanatory variable $X_{M}$ being random together with the response variable *X*, and write a pseudodistance between a theoretical model and the data. Let $P_{\theta }$, with θ=:(α,β,σ), be the probability measure associated with the theoretical model given by the random vector $\left(X_{M},X\right)$, where $X=\alpha +\beta X_{M}+e$ with e∼N(0,σ), *e* independent on $X_{M}$, and *Q* the probability measure associated with the data. Denote by $p_{\theta }$, respectively, *q*, the corresponding densities. For γ>0, the pseudodistance between $P_{\theta }$ and *Q* is defined by

(14) $$\begin{array}{cc} R_{\gamma }\left(P_{\theta },Q\right):=\; & \frac{1}{\left(1+\gamma \right)}\ln\left(\int p_{\theta }^{\gamma }\left(x_{M},x\right)dP_{\theta }\left(x_{M},x\right)\right)+\frac{1}{\gamma (1+\gamma )}\ln\left(\int q^{\gamma }\left(x_{M},x\right)dQ\left(x_{M},x\right)\right)-\\ & \frac{1}{\gamma }\ln\left(\int p_{\theta }^{\gamma }\left(x_{M},x\right)dQ\left(x_{M},x\right)\right). \end{array}$$

Using the change of variables $\left(x_{M},x\right)\to \left(u,v\right):=\left(x_{M},x-\alpha -\beta x_{M}\right)$ and taking into account that $f\left(u,v\right):=p_{\theta }\left(u,v+\alpha +\beta u\right)$ is the density of $\left(X_{M},e\right)$, since $X_{M}$ and *e* are independent, we can write

(15) $$\begin{array}{c} \int p_{\theta }^{\gamma }\left(x_{M},x\right)dP_{\theta }\left(x_{M},x\right)=\int p_{M}^{\gamma +1}\left(u\right)du\cdot \int \phi_{\sigma }^{\gamma +1}\left(v\right)dv, \end{array}$$

(16) $$\begin{array}{c} \int p_{\theta }^{\gamma }\left(x_{M},x\right)dQ\left(x_{M},x\right)=\int p_{M}^{\gamma }\left(x_{M}\right)\cdot \phi_{\sigma }^{\gamma }\left(x-\alpha -\beta x_{M}\right)dQ\left(x_{M},x\right), \end{array}$$

where $p_{M}$ is the density of $X_{M}$ and $\phi_{\sigma }$ is the density of the random variable e∼N(0,σ). Then,

$$\begin{array}{cc} R_{\gamma }\left(P_{\theta },Q\right)= & \frac{1}{\left(1+\gamma \right)}\ln\left(\int p_{M}^{\gamma +1}\left(u\right)du\right)+\frac{1}{\left(1+\gamma \right)}\ln\left(\int \phi_{\sigma }^{\gamma +1}\left(v\right)dv\right)\\ & +\frac{1}{\gamma (1+\gamma )}\ln\left(\int q^{\gamma }\left(x_{M},x\right)dQ\left(x_{M},x\right)\right)\\ & -\frac{1}{\gamma }\ln\left(\int p_{M}^{\gamma }\left(x_{M}\right)\cdot \phi_{\sigma }^{\gamma }\left(x-\alpha -\beta x_{M}\right)dQ\left(x_{M},x\right)\right). \end{array}$$

Notice that the first and the third terms in the pseudodistance $R_{\gamma }\left(P_{\theta },Q\right)$ do not depend on θ and hence are not included in the minimization process. The parameter $\theta_{0}:=\left(\alpha_{0},\beta_{0},\sigma_{0}\right)$ of interest is then given by

(17) $$\begin{array}{cc} \left(\alpha_{0},\beta_{0},\sigma_{0}\right) & :=\;\arg\min_{\alpha ,\beta ,\sigma }R_{\gamma }\left(P_{\theta },Q\right)\\ & \;=\;\arg\min_{\alpha ,\beta ,\sigma }\left\{\frac{1}{\left(1+\gamma \right)}\ln\left(\int \phi_{\sigma }^{\gamma +1}\left(v\right)dv\right)-\frac{1}{\gamma }\ln\left(\int p_{M}^{\gamma }\left(x_{M}\right)\cdot \phi_{\sigma }^{\gamma }\left(x-\alpha -\beta x_{M}\right)dQ\left(x_{M},x\right)\right)\right\}. \end{array}$$

Suppose now that an i.i.d. sample $Z_{1},\ldots ,Z_{n}$ is available from the true model. For a given γ>0, we define a minimum pseudodistance estimator of $\theta_{0}=\left(\alpha_{0},\beta_{0},\sigma_{0}\right)$ by minimizing an empirical version of the objective function in Equation (17). This empirical version is obtained by replacing $p_{M}\left(x_{M}\right)$ with the empirical density function ${\hat{p}}_{M}\left(x_{M}\right)=\frac{1}{n}\sum_{i=1}^{n}\delta \left(x_{M}-X_{Mi}\right)$, where δ(·) is the Dirac delta function, and *Q* with the empirical measure corresponding to the sample. More precisely, we define $\hat{\theta }:=\left(\hat{\alpha },\hat{\beta },\hat{\sigma }\right)$

(18) $$\begin{array}{cc} \left(\hat{\alpha },\hat{\beta },\hat{\sigma }\right) & :=\arg\min_{\alpha ,\beta ,\sigma }\left\{\frac{1}{\left(1+\gamma \right)}\ln\left(\int \phi_{\sigma }^{\gamma +1}\left(v\right)dv\right)-\frac{1}{\gamma }\ln\left(\int {\hat{p}}_{M}^{\gamma }\left(x_{M}\right)\cdot \phi_{\sigma }^{\gamma }\left(x-\alpha -\beta x_{M}\right)dP_{n}\left(x_{M},x\right)\right)\right\}\\ & \;=\arg\min_{\alpha ,\beta ,\sigma }\left\{\frac{1}{\left(1+\gamma \right)}\ln\left(\int \phi_{\sigma }^{\gamma +1}\left(v\right)dv\right)-\frac{1}{\gamma }\ln\left(\frac{1}{n^{\gamma +1}}\sum_{j=1}^{n}\phi_{\sigma }^{\gamma }\left(X_{j}-\alpha -\beta X_{Mj}\right)\right)\right\}, \end{array}$$

or equivalently

$$\begin{array}{ccc} \left(\hat{\alpha },\hat{\beta },\hat{\sigma }\right) & = & \arg\max_{\alpha ,\beta ,\sigma }\sum_{j=1}^{n}\frac{\phi_{\sigma }^{\gamma }\left(X_{j}-\alpha -\beta X_{Mj}\right)}{{\left[\int \phi_{\sigma }^{\gamma +1}\left(v\right)dv\right]}^{\gamma /(\gamma +1)}}\\ & = & \arg\max_{\alpha ,\beta ,\sigma }\sum_{j=1}^{n}\sigma^{-\gamma /(\gamma +1)}\exp\left(-\frac{\gamma }{2}{\left(\frac{X_{j}-\alpha -\beta X_{Mj}}{\sigma }\right)}^{2}\right). \end{array}$$

Differentiating with respect to α,β,σ, the estimators $\hat{\alpha },\hat{\beta },\hat{\sigma }$ are solutions of the system

(19) $$\begin{array}{c} \sum_{j=1}^{n}\exp\left(-\frac{\gamma }{2}{\left(\frac{X_{j}-\alpha -\beta X_{Mj}}{\sigma }\right)}^{2}\right)\left(\frac{X_{j}-\alpha -\beta X_{Mj}}{\sigma }\right)=0, \end{array}$$

(20) $$\begin{array}{c} \sum_{j=1}^{n}\exp\left(-\frac{\gamma }{2}{\left(\frac{X_{j}-\alpha -\beta X_{Mj}}{\sigma }\right)}^{2}\right)\left(\frac{X_{j}-\alpha -\beta X_{Mj}}{\sigma }\right)X_{Mj}=0, \end{array}$$

(21) $$\begin{array}{c} \sum_{j=1}^{n}\exp\left(-\frac{\gamma }{2}{\left(\frac{X_{j}-\alpha -\beta X_{Mj}}{\sigma }\right)}^{2}\right)\left[{\left(\frac{X_{j}-\alpha -\beta X_{Mj}}{\sigma }\right)}^{2}-\frac{1}{\gamma +1}\right]=0. \end{array}$$

Note that, for γ=0, the solution of this system is nothing but the maximum likelihood estimator of (α,β,σ). Therefore, the estimating Equations (19)–(21) are generalizations of the maximum likelihood score equations. The tuning parameter γ associated with the pseudodistance controls the trade-off between robustness and efficiency of the minimum pseudodistance estimators.

We can also write that $\hat{\theta }=\left(\hat{\alpha },\hat{\beta },\hat{\sigma }\right)$ is a solution of

(22) $$\sum_{j=1}^{n}\Psi \left(Z_{j},\hat{\theta }\right)=0\;\;\mathrm{or}\;\;\int \Psi \left(z,\hat{\theta }\right)dP_{n}\left(z\right)=0,$$

where

(23) $$\Psi \left(z,\theta \right)={\left(\phi \left(\frac{x-\alpha -\beta x_{M}}{\sigma }\right),\phi \left(\frac{x-\alpha -\beta x_{M}}{\sigma }\right)x_{M},\chi \left(\frac{x-\alpha -\beta x_{M}}{\sigma }\right)\right)}^{T},$$

with $z=(x_{M},x)$, θ=(α,β,σ), $\phi \left(t\right)=\exp\left(-\frac{\gamma }{2}t^{2}\right)t$ and $\chi \left(t\right)=\exp\left(-\frac{\gamma }{2}t^{2}\right)\left[t^{2}-\frac{1}{\gamma +1}\right]$.

When the measure *Q* corresponding to the data pertain to the theoretical model, hence $Q=P_{\theta_{0}}$, it holds that

(24) $$\int \Psi \left(z,\theta_{0}\right)dP_{\theta_{0}}\left(z\right)=0.$$

Thus, we can consider $\hat{\theta }=\left(\hat{\alpha },\hat{\beta },\hat{\sigma }\right)$ as a *Z*-estimator of $\theta_{0}=\left(\alpha_{0},\beta_{0},\sigma_{0}\right)$, which allows for adapting in the present context asymptotic results from the general theory of *Z*-estimators (see [21]).

**Remark** **1.** *In the case when the density $p_{M}$ is known, by replacing Q with the empirical measure $P_{n}$ in Equation (17), a new class of estimators of $\left(\alpha_{0},\beta_{0},\sigma_{0}\right)$ can be obtained. These estimators can also be written under the form of Z-estimators, using the same reasoning as above. The results of Theorems 1–4 below could be adapted for these new estimators, and moreover all the influence functions of these estimators would be redescending bounded. However, in practice, the density of the index return is not known. Therefore, we will work with the class of minimum pseudodistance estimators as defined above.*

### 3.2. Asymptotic Properties

In order to prove the consistency of the estimators, we use their definition (22) as *Z*-estimators.

#### 3.2.1. Consistency

**Theorem** **1.** *Assume that, for any ε>0, the following condition for the separability of solution holds*

(25) $$\underset{\theta \in M}{\inf}∥\int \psi \left(z,\theta \right)dP_{\theta_{0}}\left(z\right)∥>0=∥\int \psi \left(z,\theta_{0}\right)dP_{\theta_{0}}\left(z\right)∥,$$

*where $M:=\{\theta \;s.t.\;∥\theta -\theta_{0}∥\geq \varepsilon \}$. Then, $\hat{\theta }=\left(\hat{\alpha },\hat{\beta },\hat{\sigma }\right)$ converges in probability to $\theta_{0}=\left(\alpha_{0},\beta_{0},\sigma_{0}\right)$.*

#### 3.2.2. Asymptotic Normality

Assume that $Z_{1},\ldots ,Z_{n}$ are i.i.d. two-dimensional random vectors having the common probability distribution $P_{\theta_{0}}$. For γ>0 fixed, let $\hat{\theta }=\left(\hat{\alpha },\hat{\beta },\hat{\sigma }\right)$ be a sequence of estimators of the unknown parameter $\theta_{0}=\left(\alpha_{0},\beta_{0},\sigma_{0}\right)$, solution of

(26) $$\sum_{j=1}^{n}\Psi \left(Z_{j},\hat{\theta }\right)=0,$$

where

(27) $$\Psi \left(z,\theta \right)={\left(\sigma^{2}\phi \left(\frac{x-\alpha -\beta x_{M}}{\sigma }\right),\sigma^{2}\phi \left(\frac{x-\alpha -\beta x_{M}}{\sigma }\right)x_{M},\sigma^{2}\chi \left(\frac{x-\alpha -\beta x_{M}}{\sigma }\right)\right)}^{T},$$

with $z=(x_{M},x)$, θ=(α,β,σ), $\phi \left(t\right)=\exp\left(-\frac{\gamma }{2}t^{2}\right)t$ and $\chi \left(t\right)=\exp\left(-\frac{\gamma }{2}t^{2}\right)\left[t^{2}-\frac{1}{\gamma +1}\right]$. Note that the estimators $\hat{\theta }=\left(\hat{\alpha },\hat{\beta },\hat{\sigma }\right)$ defined by Equations (19)–(21), or equivalently by (22), are also solutions of the system (26). Using the function (27) for defining the estimators allows for obtaining the asymptotic normality, only imposing the consistency condition of the estimators, without other supplementary assumptions that are usually imposed in the case of *Z*-estimators.

**Theorem** **2.** *Assume that $\hat{\theta }\to \theta_{0}$ in probability. Then,*

(28) $$\sqrt{n}\left(\hat{\theta }-\theta_{0}\right)\to N_{3}\left(0,B^{-1}A{\left(B^{-1}\right)}^{T}\right)$$

*in distribution, where $A=E(\Psi \left(Z,\theta_{0}\right)\Psi {\left(Z,\theta_{0}\right)}^{T})$ and $B=E(\dot{\Psi }\left(Z,\theta_{0}\right))$, with Ψ defined by (27), $\dot{\Psi }$ being the matrix with elements ${\dot{\Psi }}_{ik}=\frac{\partial \Psi_{i}}{\partial \theta_{k}}$.*

After some calculations, we obtain the asymptotic covariance matrix of $\hat{\theta }$ having the form

$$\sigma_{0}^{2}\frac{{\left(\gamma +1\right)}^{3}}{{\left(2\gamma +1\right)}^{3/2}}\left(\begin{array}{ccc} \frac{\mu_{M}^{2}+\sigma_{M}^{2}}{\sigma_{M}^{2}} & \frac{-\mu_{M}}{\sigma_{M}^{2}} & 0\\ \frac{-\mu_{M}}{\sigma_{M}^{2}} & \frac{1}{\sigma_{M}^{2}} & 0\\ 0 & 0 & \frac{3\gamma^{2}+4\gamma +2}{4(2\gamma +1)} \end{array}\right).$$

It follows that $\hat{\beta }$ and $\hat{\sigma }$ are asymptotically independent; in addition, $\hat{\alpha }$ and $\hat{\sigma }$ are asymptotically independent.

### 3.3. Influence Functions

In order to describe stability properties of the estimators, we use the following well-known concepts from the theory of robust statistics. A map *T*, defined on a set of probability measures and parameter space valued, is a statistical functional corresponding to an estimator $\hat{\theta }$ of the parameter θ, if $\hat{\theta }=T\left(P_{n}\right)$, $P_{n}$ being the empirical measure pertaining to the sample. The influence function of *T* at $P_{\theta }$ is defined by

$$\mathrm{IF}\left(z;T,P_{\theta }\right):={\left.\frac{\partial T({\tilde{P}}_{\varepsilon z})}{\partial \varepsilon }\right|}_{\varepsilon =0},$$

where ${\tilde{P}}_{\varepsilon z}:=\left(1-\varepsilon \right)P_{\theta }+\varepsilon \delta_{z},$ $\delta_{z}$ being the Dirac measure putting all mass at *z*. As a consequence, the influence function describes the linearized asymptotic bias of a statistic under a single point contamination of the model $P_{\theta }$. An unbounded influence function implies an unbounded asymptotic bias of a statistic under single point contamination of the model. Therefore, a natural robustness requirement on a statistical functional is the boundedness of its influence function.

For γ>0 fixed and a given probability measure *P*, the statistical functionals α(P), β(P) and σ(P), corresponding to the minimum pseudodistance estimators $\hat{\alpha }$, $\hat{\beta }$ and $\hat{\sigma }$, are defined by the solution of the system

(29) ∫Ψ(z,T(P))dP(z)=0,

with Ψ defined by (23) and T(P):=(α(P),β(P),σ(P)), whenever this solution exists.

When $P=P_{\theta }$ corresponds to the considered theoretical model, the solution of system (29) is $T\left(P_{\theta }\right)=\theta =\left(\alpha ,\beta ,\sigma \right)$.

**Theorem** **3.** *The influence functions corresponding to the estimators $\hat{\alpha },\hat{\beta }$ and $\hat{\sigma }$ are respectively given by*

(30) $$\begin{array}{ccc} IF(x_{M0},x_{0};\alpha ,P_{\theta }) & = & \sigma {\left(\gamma +1\right)}^{3/2}\phi \left(\frac{x_{0}-\alpha -\beta x_{M0}}{\sigma }\right)\left[1-\frac{\left(x_{M0}-E\left(X_{M}\right)\right)E\left(X_{M}\right)}{Var(X_{M})}\right], \end{array}$$

(31) $$\begin{array}{ccc} IF(x_{M0},x_{0};\beta ,P_{\theta }) & = & \sigma {\left(\gamma +1\right)}^{3/2}\phi \left(\frac{x_{0}-\alpha -\beta x_{M0}}{\sigma }\right)\frac{x_{M0}-E\left(X_{M}\right)}{Var(X_{M})}, \end{array}$$

(32) $$\begin{array}{ccc} IF(x_{M0},x_{0};\sigma ,P_{\theta }) & = & \frac{\sigma {\left(\gamma +1\right)}^{5/2}}{2}\chi \left(\frac{x_{0}-\alpha -\beta x_{M0}}{\sigma }\right). \end{array}$$

Since χ is redescending, $\hat{\sigma }$ has a bounded influence function and hence it is a redescending B-robust estimator. On the other hand, $IF(x_{M0},x_{0},\alpha ,P)$ and $IF(x_{M0},x_{0},\beta ,P)$ will tend to infinity only when $x_{M0}$ tends to infinity and $|\frac{x_{0}-\alpha -\beta x_{M0}}{\sigma }|\leq k$, for some *k*. Hence, these influence functions are bounded with respect to partial outliers or leverage points (outlying values of the independent variable). This means that large outliers with respect to $x_{M}$, or with respect to *x*, will have a reduced influence on the estimates. However, the influence functions are clearly unbounded for γ=0, which corresponds to the non-robust maximum likelihood estimators.

### 3.4. Equivariance of the Regression Coefficients’ Estimators

If an estimator is equivariant, it means that it transforms "properly" in some sense. Rousseeuw and Leroy [22] (p. 116) discuss three important equivariance properties for a regression estimator: regression equivariance, scale equivariance and affine equivariance. These are desirable properties since they allow one to know how the estimates change under different types of transformations of the data. Regression equivariance means that any additional linear dependence is reflected in the regression vector accordingly. The regression equivariance is routinely used when studying regression estimators. It allows for assuming, without loss generality, any value for the parameter (α,β) for proving asymptotic properties or describing Monte-Carlo studies. An estimator being scale equivariant means that the fit produced by it is independent of the choice of measurement unit for the response variable. The affine equivariance is useful because it means that changing to a different co-ordinate system for the explanatory variable will not affect the estimate. It is known that the maximum likelihood estimator of the regression coefficients satisfies all these three properties. We show that the minimum pseudodistance estimators of the regression coefficients satisfy all the three equivariance properties, for all γ>0.

**Theorem** **4.** *For all γ>0, the minimum pseudodistance estimators ${\left(\hat{\alpha },\hat{\beta }\right)}^{T}$ of the regression coefficients ${\left(\alpha ,\beta \right)}^{T}$ are regression equivariant, scale equivariant and affine equivariant.*

On the other hand, the objective function in the definition of the estimators depends on data only through the summation

(33) $$\sum_{j=1}^{n}\sigma^{-\gamma /(\gamma +1)}\exp\left(-\frac{\gamma }{2}{\left(\frac{X_{j}-\alpha -\beta X_{Mj}}{\sigma }\right)}^{2}\right),$$

which is permutation invariant. Thus, the corresponding estimators of the regression coefficients and of the error standard deviation are permutation invariant, therefore the ordering of data does not affect the estimators.

The minimum pseudodistance estimators are also equivariant with respect to reparametrizations. If θ=(α,β,σ) and the model is reparametrized to Υ=Υ(θ) with a one-to-one transformation, then the minimum pseudodistance estimator of Υ is simply $\hat{\Upsilon }=\Upsilon \left(\hat{\theta }\right)$, in terms of the minimum pseudodistance estimator $\hat{\theta }$ of θ, for the same γ.

### 3.5. Robust Portfolios Using Minimum Pseudodistance Estimators

The robust estimation of the parameters $\alpha_{i},\beta_{i},\sigma_{i}$ from the single index model given by (35), using minimum pseudodistance estimators, together with the robust estimation of $\mu_{M}$ and $\sigma_{M}$ lead to robust estimates of μ and Σ, on the basis of relations (9)–(11). Since we do not model the explanatory variable $X_{M}$ in a specific way, we estimate $\mu_{M}$ and the standard deviation $\sigma_{M}$ using as robust estimators the median, respectively the median absolute deviation. Then, the portfolio weights, obtained as solutions of the optimization problems (4) or (7) with input parameters robustly estimated, will also be robust. This methodology leads to new optimal robust portfolios. In the next section, on the basis of real financial data, we illustrate this new methodology and compare it with the traditional method based on maximum likelihood estimators.

## 4. Applications

### 4.1. Comparisons of the Minimum Pseudodistance Estimators with Other Robust Estimators for the Linear Regression Model

In order to illustrate the performance of the minimum pseudodistance estimators for the simple linear regression model, we compare them with the least median of squares (LMS) estimator (see [22,23]), with S-estimators (SE) (see [24]) and with the minimum density power divergence (MDPD) estimators (see [25]), estimators that are known to have a good behavior from the robustness point of view.

We considered a data set that comes from astronomy, namely the data from the Hertzsprung–Russell diagram of the star clusters CYG OB1 containing 47 stars in the direction of Cygnus. For these data, the independent variable is the logarithm of the effective temperature at the surface of the star and the dependent variable is the logarithm of its light intensity. The data are given in Rousseeuw and Leroy [22] (p. 27), who underlined that there are two groups of points: the majority, following a steep band, and four stars clearly forming a separate group from the rest of the data. These four stars are known as giants in astronomy. Thus, these outliers are not recording errors, but represents leverage points coming from a different group.

**Figure 1:**
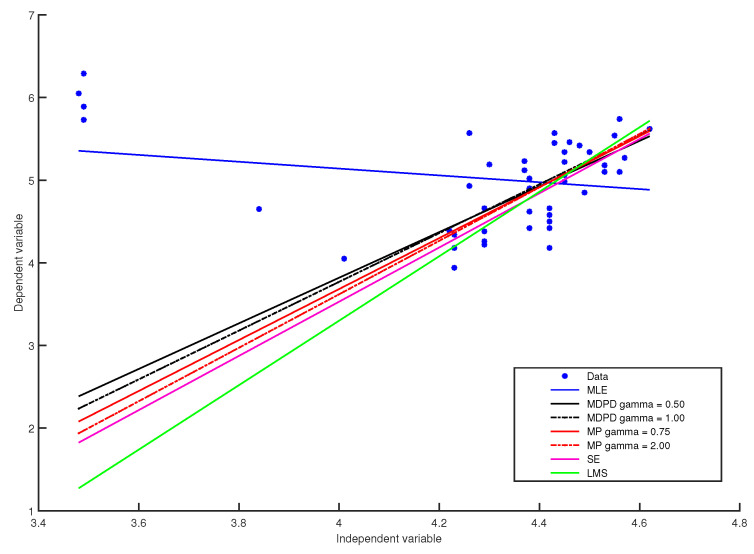
Plots of the Hertzsprung–Russell data and fitted regression lines using MLE, minimum density power divergence (MDPD) methods for several values of γ, minimum pseudodistance (MP) methods for several values of γ, S-estimators (SE) and the least median of squares (LMS) method.

The estimates of the regression coefficients and of error standard deviation obtained with minimum pseudodistance estimators for several values of γ are given in Table 1 and some of the fitted models are plotted in Figure 1. For comparison, in Table 1, we also give estimates obtained with S-estimators based on the Tukey biweighted function, these estimates being taken from [24], as well as estimations obtained with minimum density power divergence methods for several values of the tuning parameter, and estimates obtained with the least median of squares method, all these estimates being taken from [25]. The MLE estimates, given on the first line of Table 1, are significantly affected by the four leverage points. On the other hand, like the robust least median of squares estimator, the robust S-estimators and some minimum density power divergence estimators, the minimum pseudodistance estimators with γ≥0.32 can successfully ignore outliers. In addition, the minimum pseudodistance estimators with γ≥0.5 give robust fits that are closer to the fits generated by the least median of squares estimates or by the S-estimates than the fits generated by the minimum density power divergence estimates.

### 4.2. Robust Portfolios Using Minimum Pseudodistance Estimators

In order to illustrate the performance of the proposed robust portfolio optimization method, we considered real data sets for the Russell 2000 index and for 50 stocks from its components. The stocks are listed in Appendix B. We selected daily return data for the Russell 2000 index and for all these stocks from 2 January 2013 to 30 June 2016. The data were retrieved from Yahoo Finance.

The data has been divided by quarter, in total 14 quarters for index and each stock. For each quarter, on the basis of data corresponding to the index, we estimated $\mu_{M}$ and the standard deviation $\sigma_{M}$ using as robust estimators the median (MED), respectively the median absolute deviation (MAD) defined by

(34) $$MAD:=\frac{1}{0.6745}\cdot MED(|X_{i}-MED\left(X_{i}\right)\left|\right).$$

We also estimated $\mu_{M}$ and $\sigma_{M}$ classically, using sample mean and sample variance. Then, for each quarter and each of the 50 stocks, we estimated α, β and σ from the regression model using robust minimum pseudodistance estimators, respectively the classical MLE estimators. Then, on the basis of relations (9), (10) and (11), we estimated μ and Σ first using the robust estimates and then the classical estimates, all being previously computed.

Once the input parameters for the portfolio optimization procedure were estimated, for each quarter, we determined efficient frontiers, for both robust estimates and classical estimates. In both cases, the efficient frontier is determined as follows. Firstly, the range of returns is determined as the interval comprised between the return of the portfolio of global minimum risk (variance) and the maximum value of the return of a feasible portfolio, where the feasible region is

$$X=\left\{w\in R^{N}\left|w^{T}e_{N}=1,w_{k}\geq 0,\;k\in \left\{1,\ldots ,50\right\}\right.\right\}$$

and N=50. We trace each efficient frontier in 100 points; therefore, the range of returns is divided, in each case, in ninety-nine sub-intervals with

$$\mu_{1}<\mu_{2}<\cdots <\mu_{100},$$

where $\mu_{1}$ is the return of the portfolio of global minimum variance and $\mu_{100}$ is the maximum return for the feasible region *X*. We determined $\mu_{1}$ and $\mu_{100}$ using robust estimates of μ and Σ (for the robust frontier) and then using classical estimates (for the classical frontier). In each case, 100 optimization problems are solved:

$$\begin{array}{c} \arg\min_{w\in R^{N}}S\left(w\right)\\ w_{k}\geq 0,\;k\in \left\{1,\ldots ,50\right\}\\ w^{T}e_{N}=1\\ R\left(w\right)\geq \mu_{i}, \end{array}$$

where $i\in \left\{1,\ldots ,100\right\}$.

**Figure 2:**
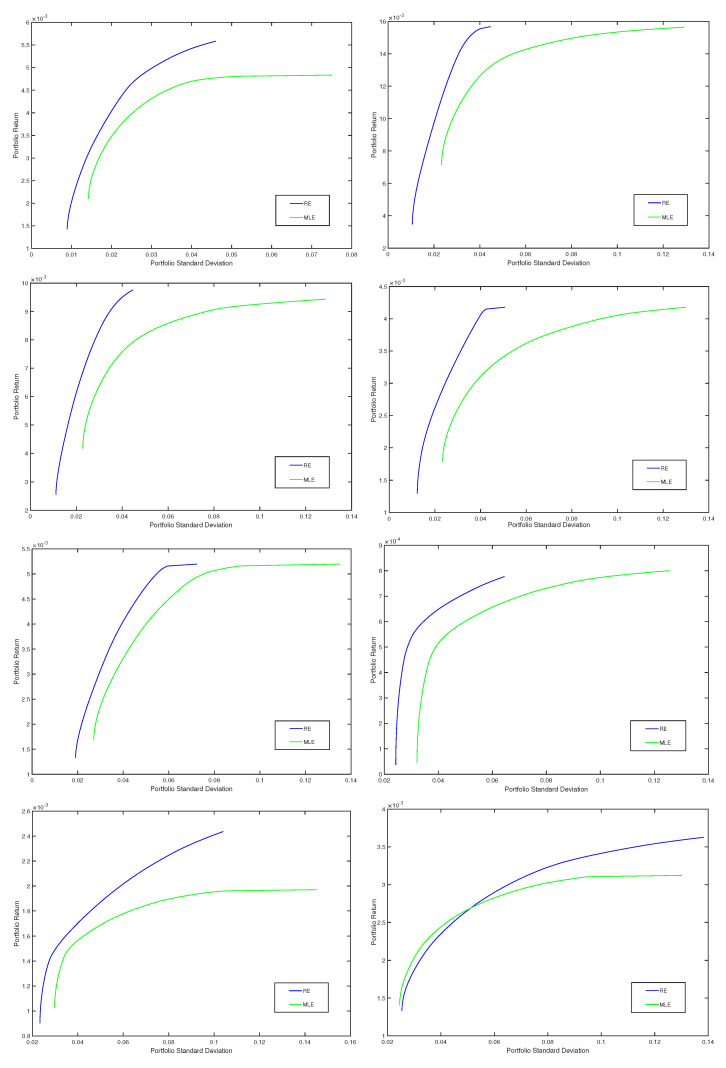
Efficient frontiers, classical (MLE) vs. robust corresponding to γ=0.5 (RE), for eight quarters (the first four quarters and the last four quarters).

In Figure 2, for eight quarters (the first four quarters and the last four quarters), we present efficient frontiers corresponding to the optimal minimum variance portfolios based on the robust minimum pseudodistance estimates with γ=0.5, respectively based on the classical estimates. Thus, on the ox-axis, we consider the portfolio risk (given by the portfolio standard deviation) and, on the oy-axis, we represent the portfolio return. We notice that, in comparison with the classical method based on MLE, the proposed robust method provides optimal portfolios that have higher returns for the same level of risk (standard deviation). Indeed, for each quarter, the robust frontier is situated above the classical one, the standard deviations of the robust portfolios being smaller compared with those of the classical portfolios. We obtained similar results for the other quarters and for other choices of the tuning parameter γ, corresponding to the minimum pseudodistance estimators, too.

We also illustrate the empirical performance of the proposed optimal portfolios through an out-of-sample analysis, by using the Sharpe ratio as out-of-sample measure. For this analysis, we apply a “rolling-horizon” procedure as presented in [18]. First, we choose a window over which to perform the estimation. We denote the length of the estimation window by τ<T, where *T* is the size of the entire data set. Then, using the data in the first estimation window, we compute the weights for the considered portfolios. We repeat this procedure for the next window, by including the data for the next day and dropping the data for the earliest day. We continue doing this until the end of the data set is reached. At the end of this process, we have generated T−τ portfolio weight vectors for each strategy, which are the vectors $w_{t}^{k}$ for t∈{τ,…,T−1}, *k* denoting the strategy. For a strategy *k*, $w_{t}^{k}$ has the components $w_{j,t}^{k}$, where $w_{j,t}^{k}$ denotes the portfolio weight in asset *j* chosen at the time *t*.

The out-of-sample return at the time t+1, corresponding to the strategy *k*, is defined as ${\left(w_{t}^{k}\right)}^{T}X_{t+1}$, $X_{t+1}:={\left(X_{1,t+1},\ldots ,X_{N,t+1}\right)}^{T}$ representing the data at the time t+1. For each strategy *k*, using these out-of-sample returns, the out-of-sample mean and the out-of-sample variance are defined by

(35) $${\hat{\mu }}^{k}=\frac{1}{T-\tau }\sum_{t=\tau }^{T-1}{\left(w_{t}^{k}\right)}^{T}X_{t+1}\;\;\;\mathrm{and}\;\;\;{\left({\hat{\sigma }}^{k}\right)}^{2}=\frac{1}{T-\tau -1}\sum_{t=\tau }^{T-1}{\left({\left(w_{t}^{k}\right)}^{T}X_{t+1}-{\hat{\mu }}^{k}\right)}^{2}$$

and the out-of-sample Sharpe ratio is defined by

(36) $${\hat{SR}}^{k}=\frac{{\hat{\mu }}^{k}}{{\hat{\sigma }}^{k}}.$$

**Figure 3:**
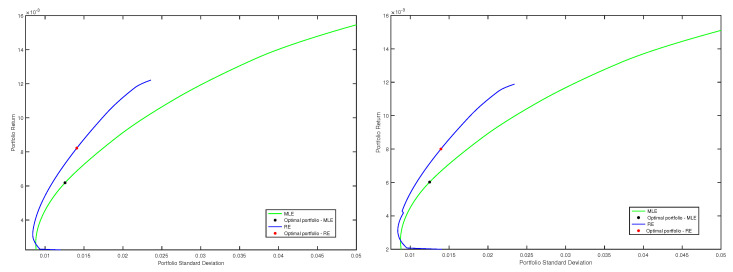
Efficient frontiers, classical (MLE) vs. robust corresponding to γ=1 (RE), and optimal portfolios chosen on frontiers, for the windows 7 (**left**) and 8 (**right**).

In this example, we considered the data set corresponding to the quarters 13 and 14. The size of the entire data set was T=126 and the length of the estimation window was τ=63 points. For the data from the first window, classical and robust efficient frontiers were traced, following all the steps that we explained in the first part of this subsection. More precisely, we considered the classical efficient frontier corresponding to the optimal minimum variance portfolios based on MLE and three robust frontiers, corresponding to the optimal minimum variance portfolios using robust minimum pseudodistance estimations with γ=1, γ=1.2 and γ=1.5, respectively. Then, on each frontier, we chose the optimal portfolio associated with the maximal value of the ratio between the portfolio return and portfolio standard deviation. These four optimal portfolios represent the strategies that we compared in the out-of-sample analysis. For each of these portfolios, we computed the out-of-sample returns for the next time (next day). Then, we repeated all these procedures for the next window, and so on until the end of the data set has been reached. In the spirit of [18] Section 5, using (35) and (36), we computed out-of-sample means, out-of-sample variances and out-of-sample Sharpe ratios for each strategy. The out-of-sample means and out-of-sample variances were annualized, and we also considered a benchmark rate of 1.5 %. In this way, we obtained the following values for the out-of-sample Sharpe ratio: $\hat{SR}=0.22$ for the optimal portfolio based on MLE, $\hat{SR}=0.74$ for the optimal portfolio based on minimum pseudodistance estimations with γ=1, $\hat{SR}=0.71$ for the optimal portfolio based on minimum pseudodistance estimations with γ=1.2 and $\hat{SR}=0.29$ for the optimal portfolio based on minimum pseudodistance estimations with γ=1.5. In Figure 3, we illustrate efficient frontiers for the windows 7 and 8, as well as the optimal portfolios chosen on each frontier.

This example shows that the optimal minimum variance portfolios based on robust minimum pseudodistance estimations in the single index model may attain higher Sharpe ratios than the traditional optimal minimum variance portfolios given by the single index model using MLE.

The obtained numerical results show that, for the single index model, the presented robust technique for portfolio optimization yields better results than the classical method based on MLE, in the sense that it leads to larger returns for the same value of risk in the case when outliers or atypical observations are present in the data set. The considered data sets contain such outliers. This is often the case for the considered problem, since outliers frequently occur in asset returns data. However, when there are no outliers in the data set, the classical method based on MLE is more efficient than the robust ones and therefore may lead to better results.

## 5. Conclusions

When outliers or atypical observations are present in the data set, the new portfolio optimization method based on robust minimum pseudodistance estimates yields better results than the classical single index method based on MLE estimates, in the sense that it leads to larger returns for smaller risks. In literature, there exist various methods for robust estimation in regression models. In the present paper, we proposed the method based on the minimum pseudodistance approach, which suppose to solve a simple optimization problem. In addition, from a theoretical point of view, these estimators have attractive properties, such as being redescending robust, consistent, equivariant and asymptotically normally distributed. The comparison with other known robust estimators of the regression parameters, such as the least median of squares estimators, the S-estimators or the minimum density power divergence estimators, shows that the minimum pseudodistance estimators represent an attractive alternative that may be considered in other applications too.

## Figures and Tables

**Table 1:** The parameter estimates for the linear regression model for the Hertzsprung–Russell data using several minimum pseudodistance (MP) methods, several minimum density power divergence (MDPD) methods, the least median of squares (LMS) method, S-estimators and the MLE method. γ represents tuning parameter.

**MLE Estimates**			
	α	β	σ
	6.79	−0.41	0.55
**MP Estimates**			
γ	α	β	σ
0.01	6.79	−0.41	0.55
0.1	6.81	−0.41	0.56
0.25	6.86	−0.42	0.58
0.3	6.88	−0.42	0.59
0.31	6.89	−0.43	0.59
0.32	−6.81	2.66	0.39
0.35	−7.16	2.74	0.38
0.4	−7.62	2.85	0.38
0.5	−8.17	2.97	0.37
0.75	−8.65	3.08	0.38
1	−8.84	3.12	0.39
1.2	−8.94	3.15	0.40
1.5	−9.08	3.18	0.41
2	−9.31	3.23	0.43
**MDPD Estimates**			
γ	α	β	σ
0.1	6.78	−0.41	0.60
0.25	−5.16	2.30	0.42
0.5	−7.22	2.76	0.40
0.8	−7.89	2.91	0.40
1	−8.03	2.95	0.41
**S-Estimates**			
	α	β	σ
	−9.59	3.28	−
**LMS Estimates**			
	α	β	σ
	−12.30	3.90	−

## Appendix A. Proof of the Results

**Proof of Theorem** **1.** Since the functions ϕ and χ are redescending bounded functions, for a compact neighborhood $N_{\theta_{0}}$ of $\theta_{0}$, it holds

(A1) $$\int \underset{\theta \in N_{\theta_{0}}}{\sup}∥\Psi \left(z,\theta \right)∥dP_{\theta_{0}}\left(z\right)<\infty .$$

Since θ↦Ψ(z,θ) is continuous, by the uniform law of large numbers, (A1) implies

(A2) $$\underset{\theta \in N_{\theta_{0}}}{\sup}∥\int \psi \left(z,\theta \right)dP_{n}\left(z\right)-\int \psi \left(z,\theta \right)dP_{\theta_{0}}\left(z\right)∥\to 0$$

in probability. Then, (A2) together with assumption (25) assure the convergence in probability of $\hat{\theta }$ toward $\theta_{0}$. The arguments are the same as those from van der Vaart [21], Theorem 5.9, p. 46. ☐

**Proof of Theorem** **2.** First, note that Ψ defined by (27) is twice differentiable with respect to θ with bounded derivatives. The matrix $\dot{\Psi }\left(z,\theta \right)$ has the form

$$\left(\begin{array}{ccc} -\sigma \phi^{\prime }\left(\frac{x-\alpha -\beta x_{M}}{\sigma }\right) & -\sigma \phi^{\prime }\left(\frac{x-\alpha -\beta x_{M}}{\sigma }\right)x_{M} & 2\sigma \phi \left(\frac{x-\alpha -\beta x_{M}}{\sigma }\right)-\sigma \phi^{\prime }\left(\frac{x-\alpha -\beta x_{M}}{\sigma }\right)\left(\frac{x-\alpha -\beta x_{M}}{\sigma }\right)\\ -\sigma \phi^{\prime }\left(\frac{x-\alpha -\beta x_{M}}{\sigma }\right)x_{M} & -\sigma \phi^{\prime }\left(\frac{x-\alpha -\beta x_{M}}{\sigma }\right)x_{M}^{2} & 2\sigma \phi \left(\frac{x-\alpha -\beta x_{M}}{\sigma }\right)x_{M}-\sigma \phi^{\prime }\left(\frac{x-\alpha -\beta x_{M}}{\sigma }\right)\left(\frac{x-\alpha -\beta x_{M}}{\sigma }\right)x_{M}\\ -\sigma \chi^{\prime }\left(\frac{x-\alpha -\beta x_{M}}{\sigma }\right) & -\sigma \chi^{\prime }\left(\frac{x-\alpha -\beta x_{M}}{\sigma }\right)x_{M} & 2\sigma \chi \left(\frac{x-\alpha -\beta x_{M}}{\sigma }\right)-\sigma \chi^{\prime }\left(\frac{x-\alpha -\beta x_{M}}{\sigma }\right)\left(\frac{x-\alpha -\beta x_{M}}{\sigma }\right) \end{array}\right)$$

with $\phi^{\prime }\left(t\right)=\left[1-\gamma t^{2}\right]\exp\left(-\frac{\gamma }{2}t^{2}\right)$ and $\chi^{\prime }\left(t\right)=\left[\frac{3\gamma +2}{\gamma +1}t-\gamma t^{3}\right]\exp\left(-\frac{\gamma }{2}t^{2}\right)$. Since $\phi \left(t\right),\chi \left(t\right),\phi^{\prime }\left(t\right),\chi^{\prime }\left(t\right)$ are redescending bounded functions, for $\theta =\theta_{0}$, it holds

(A3) $$|{\dot{\Psi }}_{ik}\left(z,\theta_{0}\right)|\leq K\left(z\right)\;\mathrm{with}\;E\left(K\left(Z\right)\right)<\infty .$$

In addition, a simple calculation shows that each component $\frac{\partial \Psi_{i}}{\partial \theta_{k}\partial \theta_{l}}$ is a bounded function, since it can be expressed through the functions $\phi \left(t\right),\chi \left(t\right),\phi^{\prime }\left(t\right),\chi^{\prime }\left(t\right),\phi^{\prime \prime }\left(t\right),\chi^{\prime \prime }\left(t\right)$, which are redescending bounded functions. In addition, bounds that can be established for each component $\frac{\partial \Psi_{i}}{\partial \theta_{k}\partial \theta_{l}}$ do not depend on the parameter θ. For each *i*, call ${\ddot{\Psi }}_{i}$ the matrix with elements $\frac{\partial \Psi_{i}}{\partial \theta_{k}\partial \theta_{l}}$ and $C_{n}\left(z,\theta \right)$ the matrix with its *i*-th raw equal to ${\left(\hat{\theta }-\theta_{0}\right)}^{T}{\ddot{\Psi }}_{i}\left(z,\theta \right)$. Using a Taylor expansion, we get

(A4) $$0=\sum_{j=1}^{n}\Psi \left(Z_{j},\hat{\theta }\right)=\sum_{j=1}^{n}\left\{\Psi \left(Z_{j},\theta_{0}\right)+\dot{\Psi }\left(Z_{j},\theta_{0}\right)\left(\hat{\theta }-\theta_{0}\right)+\frac{1}{2}C_{n}\left(Z_{j},\theta_{j}\right)\left(\hat{\theta }-\theta_{0}\right)\right\}.$$

Therefore,

(A5) $$0=A_{n}+\left(B_{n}+{\bar{C}}_{n}\right)\left(\hat{\theta }-\theta_{0}\right)$$

with

(A6) $$A_{n}=\frac{1}{n}\sum_{j=1}^{n}\Psi \left(Z_{j},\theta_{0}\right),\;B_{n}=\frac{1}{n}\sum_{j=1}^{n}\dot{\Psi }\left(Z_{j},\theta_{0}\right),\;{\bar{C}}_{n}=\frac{1}{2n}\sum_{j=1}^{n}C_{n}\left(Z_{j},\theta_{j}\right)$$

i.e., ${\bar{C}}_{n}$ is the matrix with its *i*-th raw equal to ${\left(\hat{\theta }-\theta_{0}\right)}^{T}{\ddot{\Psi }}_{i}^{-}$, where

(A7) $${\ddot{\Psi }}_{i}^{-}=\frac{1}{2n}\sum_{j=1}^{n}{\ddot{\Psi }}_{i}\left(Z_{j},\theta_{j}\right),$$

which is bounded by a constant that does not depend on θ, according to the arguments mentioned above. Since $\hat{\theta }-\theta_{0}\to 0$ in probability, this implies that ${\bar{C}}_{n}\to 0$ in probability. We have

(A8) $$\sqrt{n}\left(\hat{\theta }-\theta_{0}\right)=-{\left(B_{n}+{\bar{C}}_{n}\right)}^{-1}\sqrt{n}A_{n}.$$

Note that, for j=1,…,n, the vectors $\Psi (Z_{j},\theta_{0})$ are i.i.d. with mean zero and the covariance matrix *A*, and the matrices $\dot{\Psi }\left(Z_{j},\theta_{0}\right)$ are i.i.d. with mean *B*. Hence, when n→∞, using (A3), the law of large numbers implies that $B_{n}\to B$ in probability, which implies $B_{n}+{\bar{C}}_{n}\to B$ in probability, which is nonsingular. Then, the multivariate central limit theorem implies $\sqrt{n}A_{n}\to N_{3}\left(0,A\right)$ in distribution. Then,

(A9) $$\sqrt{n}\left(\hat{\theta }-\theta_{0}\right)\to N_{3}\left(0,B^{-1}A{\left(B^{-1}\right)}^{T}\right)$$

in distribution, according to the multivariate Slutzki’s Lemma. ☐

**Proof of Theorem** **3.** The system (29) can be written as

$$\begin{array}{c} \int \phi \left(\frac{x-\alpha \left(P\right)-\beta \left(P\right)x_{M}}{\sigma (P)}\right)dP\left(x_{M},x\right)=0,\\ \int \phi \left(\frac{x-\alpha \left(P\right)-\beta \left(P\right)x_{M}}{\sigma (P)}\right)x_{M}dP\left(x_{M},x\right)=0,\\ \int \chi \left(\frac{x-\alpha \left(P\right)-\beta \left(P\right)x_{M}}{\sigma (P)}\right)dP\left(x_{M},x\right)=0. \end{array}$$

We consider the contaminated model ${\tilde{P}}_{\varepsilon ,x_{M0},x_{0}}:=\left(1-\varepsilon \right)P_{\theta }+\varepsilon \delta_{\left(x_{M0},x_{0}\right)}$, where $\delta_{\left(x_{M0},x_{0}\right)}$ is the Dirac measure putting all mass in the point $\left(x_{M0},x_{0}\right)$, which we simply denote here by ${\tilde{P}}_{\varepsilon }$. Then, it holds

(A10) $$\begin{array}{c} \left(1-\varepsilon \right)\int \phi \left(\frac{x-\alpha \left({\tilde{P}}_{\varepsilon }\right)-\beta \left({\tilde{P}}_{\varepsilon }\right)x_{M}}{\sigma ({\tilde{P}}_{\varepsilon })}\right)dP_{\theta }\left(x_{M},x\right)+\varepsilon \phi \left(\frac{x_{0}-\alpha \left({\tilde{P}}_{\varepsilon }\right)-\beta \left({\tilde{P}}_{\varepsilon }\right)x_{M0}}{\sigma ({\tilde{P}}_{\varepsilon })}\right)=0, \end{array}$$

(A11) $$\begin{array}{c} \left(1-\varepsilon \right)\int \phi \left(\frac{x-\alpha \left({\tilde{P}}_{\varepsilon }\right)-\beta \left({\tilde{P}}_{\varepsilon }\right)x_{M}}{\sigma ({\tilde{P}}_{\varepsilon })}\right)x_{M}dP_{\theta }\left(x_{M},x\right)+\varepsilon \phi \left(\frac{x_{0}-\alpha \left({\tilde{P}}_{\varepsilon }\right)-\beta \left({\tilde{P}}_{\varepsilon }\right)x_{M0}}{\sigma ({\tilde{P}}_{\varepsilon })}\right)x_{M0}=0, \end{array}$$

(A12) $$\begin{array}{c} \left(1-\varepsilon \right)\int \chi \left(\frac{x-\alpha \left({\tilde{P}}_{\varepsilon }\right)-\beta \left({\tilde{P}}_{\varepsilon }\right)x_{M}}{\sigma ({\tilde{P}}_{\varepsilon })}\right)dP_{\theta }\left(x_{M},x\right)+\varepsilon \chi \left(\frac{x_{0}-\alpha \left({\tilde{P}}_{\varepsilon }\right)-\beta \left({\tilde{P}}_{\varepsilon }\right)x_{M0}}{\sigma ({\tilde{P}}_{\varepsilon })}\right)=0. \end{array}$$

Derivating the first equation with respect to ε and taking the derivatives in ε=0, we obtain

$$\begin{array}{c} \int \phi^{\prime }\left(\frac{x-\alpha -\beta x_{M}}{\sigma }\right)\left[\frac{1}{\sigma }\left(-IF\left(x_{M0},x_{0},\alpha ,P_{\theta }\right)-x_{M}IF\left(x_{M0},x_{0},\beta ,P_{\theta }\right)\right)\right.\\ \left.-\frac{x-\alpha -\beta x_{M}}{\sigma^{2}}IF\left(x_{M0},x_{0},\sigma ,P_{\theta }\right)\right]dP_{\theta }\left(x_{M},x\right)+\phi \left(\frac{x_{0}-\alpha -\beta x_{M0}}{\sigma }\right)=0. \end{array}$$

After some calculations, we obtain the relation

(A13) $$\begin{array}{c} -\frac{1}{\sigma {\left(\gamma +1\right)}^{3/2}}IF\left(x_{M0},x_{0},\alpha ,P_{\theta }\right)-\frac{1}{\sigma {\left(\gamma +1\right)}^{3/2}}IF\left(x_{M0},x_{0},\beta ,P_{\theta }\right)E\left(X_{M}\right)+\phi \left(\frac{x_{0}-\alpha -\beta x_{M0}}{\sigma }\right)=0. \end{array}$$

Similarly, derivating with respect to ε Equations (A11) and (A12) and taking the derivatives in ε=0, we get

(A14) $$\begin{array}{c} -\frac{1}{\sigma {\left(\gamma +1\right)}^{3/2}}E\left(X_{M}\right)IF\left(x_{M0},x_{0},\alpha ,P_{\theta }\right)-\frac{1}{\sigma {\left(\gamma +1\right)}^{3/2}}E\left(X_{M}^{2}\right)IF\left(x_{M0},x_{0},\beta ,P_{\theta }\right)\\ +\phi \left(\frac{x_{0}-\alpha -\beta x_{M0}}{\sigma }\right)x_{M0}=0 \end{array}$$

and

(A15) $$-\frac{2}{\sigma {\left(\gamma +1\right)}^{5/2}}IF\left(x_{M0},x_{0},\sigma ,P_{\theta }\right)+\chi \left(\frac{x_{0}-\alpha -\beta x_{M0}}{\sigma }\right)=0.$$

Solving the system formed with the Equations (A13)–(A15), we find the expressions for the influence functions. ☐

**Proof of Theorem** **4.** In the following, we simply denote by $X_{Mj}$ the vector ${\left(1,X_{Mj}\right)}^{T}$. Then,

$$\begin{array}{c} {\left(\hat{\alpha },\hat{\beta }\right)}^{T}\left(\left\{\left(X_{Mj},X_{j}\right):j=1,\ldots ,n\right\}\right)\\ =\arg_{{\left(\alpha ,\beta \right)}^{T}}\max_{\left(\alpha ,\beta ,\sigma \right)}\sum_{j=1}^{n}\sigma^{-\gamma /(\gamma +1)}\exp\left(-\frac{\gamma }{2}{\left(\frac{X_{j}-X_{Mj}^{T}{\left(\alpha ,\beta \right)}^{T}}{\sigma }\right)}^{2}\right). \end{array}$$

For any two-dimensional column vector *v*, we have

$$\begin{array}{c} {\left(\hat{\alpha },\hat{\beta }\right)}^{T}\left(\left\{\left(X_{Mj},X_{j}+X_{Mj}^{T}v\right):j=1,\ldots ,n\right\}\right)\\ =\arg_{{\left(\alpha ,\beta \right)}^{T}}\max_{\left(\alpha ,\beta ,\sigma \right)}\sum_{j=1}^{n}\sigma^{-\gamma /(\gamma +1)}\exp\left(-\frac{\gamma }{2}{\left(\frac{X_{j}+X_{Mj}^{T}v-X_{Mj}^{T}{\left(\alpha ,\beta \right)}^{T}}{\sigma }\right)}^{2}\right)\\ =\arg_{{\left(\alpha ,\beta \right)}^{T}}\max_{\left(\alpha ,\beta ,\sigma \right)}\sum_{j=1}^{n}\sigma^{-\gamma /(\gamma +1)}\exp\left(-\frac{\gamma }{2}{\left(\frac{X_{j}-X_{Mj}^{T}\left({\left(\alpha ,\beta \right)}^{T}-v\right)}{\sigma }\right)}^{2}\right)\\ =\arg_{\left({\left(\alpha ,\beta \right)}^{T}-v\right)}\max_{{\left({\left(\alpha ,\beta \right)}^{T}-v\right)}^{T},\sigma )}\sum_{j=1}^{n}\sigma^{-\gamma /(\gamma +1)}\exp\left(-\frac{\gamma }{2}{\left(\frac{X_{j}-X_{Mj}^{T}\left({\left(\alpha ,\beta \right)}^{T}-v\right)}{\sigma }\right)}^{2}\right)+v\\ ={\left(\hat{\alpha },\hat{\beta }\right)}^{T}\left(\left\{\left(X_{Mj},X_{j}\right):j=1,\ldots ,n\right\}\right)+v, \end{array}$$

which show that ${\left(\hat{\alpha },\hat{\beta }\right)}^{T}$ is regression equivariant. For any constant c≠0, we have

$$\begin{array}{c} {\left(\hat{\alpha },\hat{\beta }\right)}^{T}\left(\left\{\left(X_{Mj},cX_{j}\right):j=1,\ldots ,n\right\}\right)\\ =\arg_{{\left(\alpha ,\beta \right)}^{T}}\max_{\left(\alpha ,\beta ,\sigma \right)}\sum_{j=1}^{n}\sigma^{-\gamma /(\gamma +1)}\exp\left(-\frac{\gamma }{2}{\left(\frac{cX_{j}-X_{Mj}^{T}{\left(\alpha ,\beta \right)}^{T}}{\sigma }\right)}^{2}\right)\\ =\arg_{{\left(\alpha ,\beta \right)}^{T}}\max_{\left(\alpha ,\beta ,\sigma \right)}\sum_{j=1}^{n}c^{-\gamma /(\gamma +1)}{\left(\sigma /c\right)}^{-\gamma /(\gamma +1)}\exp\left(-\frac{\gamma }{2}{\left(\frac{X_{j}-X_{Mj}^{T}\left({\left(\alpha ,\beta \right)}^{T}/c\right)}{\left(\sigma /c\right)}\right)}^{2}\right)\\ =c\cdot \arg_{{\left(\alpha /c,\beta /c\right)}^{T}}\max_{\left(\alpha /c,\beta /c,\sigma /c\right)}\sum_{j=1}^{n}c^{-\gamma /(\gamma +1)}{\left(\sigma /c\right)}^{-\gamma /(\gamma +1)}\exp\left(-\frac{\gamma }{2}{\left(\frac{X_{j}-X_{Mj}^{T}\left({\left(\alpha ,\beta \right)}^{T}/c\right)}{\left(\sigma /c\right)}\right)}^{2}\right)\\ =c\cdot {\left(\hat{\alpha },\hat{\beta }\right)}^{T}\left(\left\{\left(X_{Mj},X_{j}\right):j=1,\ldots ,n\right\}\right). \end{array}$$

This implies that the estimator $\left(\hat{\alpha },\hat{\beta }\right)=\left(\hat{\alpha },\hat{\beta }\right)\left(\left\{\left(X_{Mj},X_{j}\right):j=1,\ldots ,n\right\}\right)$ is scale equivariant. Now, for any two-dimensional square matrix *A*, we get

$$\begin{array}{c} {\left(\hat{\alpha },\hat{\beta }\right)}^{T}\left(\left\{\left(A^{T}X_{Mj},X_{j}\right):j=1,\ldots ,n\right\}\right)\\ =\arg_{{\left(\alpha ,\beta \right)}^{T}}\max_{\left(\alpha ,\beta ,\sigma \right)}\sum_{j=1}^{n}\sigma^{-\gamma /(\gamma +1)}\exp\left(-\frac{\gamma }{2}{\left(\frac{X_{j}-X_{Mj}^{T}A{\left(\alpha ,\beta \right)}^{T}}{\sigma }\right)}^{2}\right)\\ =A^{-1}\arg_{A{\left(\alpha ,\beta \right)}^{T}}\max_{\left(\left(\alpha ,\beta \right)A^{T},\sigma \right)}\sum_{j=1}^{n}\sigma^{-\gamma /(\gamma +1)}\exp\left(-\frac{\gamma }{2}{\left(\frac{X_{j}-X_{Mj}^{T}\left(A{\left(\alpha ,\beta \right)}^{T}\right)}{\sigma }\right)}^{2}\right)\\ =A^{-1}\cdot {\left(\hat{\alpha },\hat{\beta }\right)}^{T}\left(\left\{\left(X_{Mj},X_{j}\right):j=1,\ldots ,n\right\}\right), \end{array}$$

which show the affine equivariance of the estimator $\left(\hat{\alpha },\hat{\beta }\right)=\left(\hat{\alpha },\hat{\beta }\right)\left(\left\{\left(X_{Mj},X_{j}\right):j=1,\ldots ,n\right\}\right)$. ☐

## Appendix B. The 50 Stocks and Their Abbreviations

1. Asbury Automotive Group, Inc. (ABG)
2. Arctic Cat Inc. (ACAT)
3. American Eagle Outfitters, Inc. (AEO)
4. AK Steel Holding Corporation (AKS)
5. Albany Molecular Research, Inc. (AMRI)
6. The Andersons, Inc. (ANDE)
7. ARMOUR Residential REIT, Inc. (ARR)
8. BJ’s Restaurants, Inc. (BJRI)
9. Brooks Automation, Inc. (BRKS)
10. Caleres, Inc. (CAL)
11. Cincinnati Bell Inc. (CBB)
12. Calgon Carbon Corporation (CCC)
13. Coeur Mining, Inc. (CDE)
14. Cohen & Steers, Inc. (CNS)
15. Cray Inc. (CRAY)
16. Cirrus Logic, Inc. (CRUS)
17. Covenant Transportation Group, Inc. (CVTI)
18. EarthLink Holdings Corp. (ELNK)
19. Gray Television, Inc. (GTN)
20. Triple-S Management Corporation (GTS)
21. Getty Realty Corp. (GTY)
22. Hecla Mining Company (HL)
23. Harmonic Inc. (HLIT)
24. Ligand Pharmaceuticals Incorporated (LGND)
25. Louisiana-Pacific Corporation (LPX)
26. Lattice Semiconductor Corporation (LSCC)
27. ManTech International Corporation (MANT)
28. MiMedx Group, Inc. (MDXG)
29. Medifast, Inc. (MED)
30. Mentor Graphics Corporation (MENT)
31. Mistras Group, Inc. (MG)
32. Mesa Laboratories, Inc. (MLAB)
33. Meritor, Inc. (MTOR)
34. Monster Worldwide, Inc. (MWW)
35. Nektar Therapeutics (NKTR)
36. Osiris Therapeutics, Inc. (OSIR)
37. PennyMac Mortgage Investment Trust (PMT)
38. Paratek Pharmaceuticals, Inc. (PRTK)
39. Repligen Corporation (RGEN)
40. Rigel Pharmaceuticals, Inc. (RIGL)
41. Schnitzer Steel Industries, Inc. (SCHN)
42. comScore, Inc. (SCOR)
43. Safeguard Scientifics, Inc. (SFE)
44. Silicon Graphics International (SGI)
45. Sagent Pharmaceuticals, Inc. (SGNT)
46. Semtech Corporation (SMTC)
47. Sapiens International Corporation N.V. (SPNS)
48. Sarepta Therapeutics, Inc. (SRPT)
49. Take-Two Interactive Software, Inc. (TTWO)
50. Park Sterling Corporation (PSTB)
